# No evidence of enhanced oxidant production in blood obtained from patients with obstructive sleep apnea

**DOI:** 10.1186/1477-5751-7-10

**Published:** 2008-11-25

**Authors:** Izabela Grabska-Kobylecka, Andrzej Kobylecki, Piotr Bialasiewicz, Maciej Krol, Golsa Ehteshamirad, Marek Kasielski, Dariusz Nowak

**Affiliations:** 1 Sleep and Respiratory Disorders Center of the Chair of Experimental and Clinical Physiology, Medical University of Lodz, 92-215 Lodz, Mazowiecka St. 6/8, Poland; 2 Bases of Clinical Medicine Teaching Center, Medical University of Lodz, 90-153 Lodz, Kopcinskiego St. 20, Poland

## Abstract

**Background:**

Obstructive sleep apnea syndrome (OSAS) is a recognized risk factor for cardiovascular morbidity and mortality, perhaps due to causative exacerbations of systemic oxidative stress. Putative oxidative stress related to numerous episodes of intermittent hypoxia, may be an oxidants chief driving force in OSAS patients.

**Methods:**

We assessed the resting and n-formyl-methionyl-leucyl-phenylalanine (fMLP)- induced whole blood chemiluminescence (as a measure of oxidant production by polymorphonuclear leukocytes and monocytes), ferric reducing ability of plasma (FRAP) and H_2_O_2 _generation in the whole blood of 27 untreated OSAS patients, 22 subjects after a night of CPAP therapy and 11 controls without OSAS. All of them were matched to age, BMI (body mass index) and smoking habits. All parameters were measured before and after polysomnography-controlled sleep, individual results were obtained as a mean from duplicated experiments.

**Results:**

No significant differences were distinguished between evening and morning blood chemiluminescence, H_2_O_2 _activity and FRAP within and between all three study groups.

For instance patients with untreated OSAS had similar morning and evening resting whole blood chemiluminescence (2.3 +/- 2.2 vs. 2.4 +/- 2.2 [aU·10^-4 ^phagocytes]), total light emission after stimulation with fMLP (1790 +/- 1371 vs. 1939 +/- 1532 [aU·s·10^-4 ^phagocytes]), as well as FRAP after 3 min. plasma incubation (602 +/- 202 vs. 671 +/- 221 [uM]). Although, in the subgroup of 11 patients with severe OSAS (apnea/hypopnea index 58 +/- 18/h and oxygen desaturation index 55 +/- 19/h), the morning vs. evening resting chemiluminescence and total light emission after stimulation with fMLP observed a propensity to elevate 2.5 +/- 2.7 vs. 1.9 +/- 1.8 [aU·10^-4 ^phagocytes] and 1778 +/- 1442 vs. 1503 +/- 1391 [aU·s·10^-4 ^phagocytes], respectively, these did not attain statistical significance (p > 0.05).

**Conclusion:**

Our investigation exposed no evidence in the overproduction of oxidants via circulating phagocytes, once considered a culprit in the oxidative stress of OSAS patients.

## Background

The presence of obstructive sleep apnea syndrome (OSAS) is strongly associated with augmented morbidity and mortality from cardiovascular diseases including arterial hypertension, cardiac arrhythmias and ischemic heart disease [1,2]. OSAS is also considered to be a risk factor of stroke and sudden cardiac death [3,4]. There appears to be practicable links between OSAS and occurrences such as apnea-induced intermittent hypoxia (IH) of tissues, sympathetic over activities during sleep [5,6] as well as putative oxidative stress in relation to the systemic inflammatory response [6,7]. Primed and or activated circulating polymorphonuclear leukocytes (PMNs) and monocytes can be a source of reactive oxygen species (ROS) in OSAS patients [7]. Indeed, the in vitro incubation of whole blood under decreased partial oxygen pressure (pO_2 _≤ 46 mm Hg) resulted in the degranulation of PMNs [8] and increased ROS production [9].

Moreover, healthy volunteers subjected to 20 min hypobaric hypoxia in a decompression chamber presented with elevated production of ROS by PMNs [10].

Due to repeated apnea and/or hypopnea episodes, nocturnal PaO_2 _can fall below 50 mmHg in OSAS patients [11,12] and thus favor activation and enhance ROS production by means of blood phagocytes. Scanty and conflicting data concerning ROS production by blood phagocytes in the course of OSAS have been published so far [13-16]. Müns et al. did not establish any alterations in ROS production accompanying ingestion of *Escherichia coli *by PMNs obtained from OSAS patients [13]. Other researchers have illustrated that increasing the agonist n-formyl-methionyl-leucyl-phenylalanine (fMLP), induced production of superoxide radicals in isolated PMNs from OSAS patients [14]. However, the limitation of that study attributes to the control groups' significant difference in respect to age, body mass index (BMI) as well as habitual cigarette smoking. In addition, the control group included cancer patients and healthy subjects not matched to OSAS patients with comorbidity [14]. In another study Dyugovskaya et al. [15] identified subpopulations of monocytes and PMNs in OSAS patients (using the flow cytometry technique) producing more ROS than the cells from the control group. Yet, in this study, the control group also differed with regard to BMI, comorbidity and concomitant pharmacological treatment. On the other hand, the significant suppression of fMLP- induced respiratory burst of isolated PMNs was reported in patients with severe OSAS using the chemiluminescence technique [16-18]. Taking into account the divergences between these studies, we decided to monitor ROS production by PMNs and monocytes in both untreated- and CPAP-treated OSAS patients and age-, BMI- and cigarette-smoking habit- matched controls (volunteers without OSAS). Two additional variables reflecting oxidant/antioxidant imbalances were determined: the H_2_O_2 _activity in the whole blood [19] and the ferric reducing ability of plasma (FRAP) [20]. We found that the apnea/hypopnea episodes during sleep did not change the morning and evening intensity of the resting as well as fMLP-induced LBCL, FRAP and blood H_2_O_2 _activities in the OSAS patients. These suggest a lack of apparent ROS overproduction via circulating PMNs in OSAS patients.

## Methods

### Chemicals and solutions

Luminol, horseradish peroxidase (HRP, 222 I.U./mg), 2,4,6-tripyridyl-s-triazine (TPTZ), bovine catalase (2440 I.U./mg of solid substance), n-formyl-methionyl-leucyl-phenylalanine (fMLP), FeCl_3_·6H_2_O and dimethyl sulphoxide (DMSO) were obtained from Sigma Chemical Co. (St. Louis, MO, USA). Phenol red and phosphate buffered saline (PBS, pH 7.4) came from ICN Biomedicals Inc (Aurora, OH, USA) and Biomed (Lublin, Poland). All other reagents were of analytical grade and were purchased from the POCH (Gliwice, Poland). Stock solutions of 1.4 M luminol in 0.1 M phosphate buffer (pH 7.4), 20 mM fMLP in DMSO, catalase in 0.9% NaCl (50 I.U/μl) and HRP in 50 mM phosphate buffer (0.925 I.U/μl, pH 7.0), 10 mM TPTZ in 40 mM HCl, and 0.028 M phenol red in water were stored in single use aliquots in the dark at -70°C. fMLP was diluted just before use with 0.9% NaCl to a final concentration 0.2 mM. Sterile, deionized, pyrogen-free water for HPLC (resistance > 18 Ωcm, Water Purification System USF ELGA, Buckinghamshire, UK) was used throughout the study.

### Study population and polysomnography

Sixty patients that underwent polysomnography at the Sleep Laboratory were studied. Thirty-eight patients underwent diagnostic polysomonography following a preliminary diagnosis of OSAS. The remaining 22, previously diagnosed with OSAS met indication for continuous positive airway pressure (CPAP) treatment.

The inclusion criteria incorporated an age span from 40 to 70 years along with a written informed consent. The exclusion criteria involved pregnancy, presence of any active infectious or inflammatory process, chronic obstructive pulmonary disease (COPD), unstable angina, uncontrolled hypertension or hypertension diagnosed within the last three months, insulin-dependent diabetes, and any surgery within the last three months, or treatment with antibiotics, nonsteriodal anti-inflammatory drugs, vitamins as well as any food supplements with antioxidant potential within the preceding two weeks. All participants, except for patients treated with CPAP, underwent a standard overnight (7 hours from about 11:00 pm to 6:00 am) polysomnography (SleepLabPro, Jaeger, VIASYS Healthcare Hoechberg, Germany). This entailed an EEG, electrooculography, chin muscles as well as an anterior tibial electromyography (EMG), a unipolar EKG, snoring detection, body position, measurement of oronasal airflow along with abdominal and chest respiratory movements as previously described [21]. Participants previously diagnosed with OSAS, underwent a similar standard polysomnography trial CPAP treatment (CPAP Respironics, RemStar Plus, Murrysville, Pennsylvania, USA) along with a mask pressure monitoring as a substitute in measurement of oronasal flow. The polysomnography enabled differentiation in two groups of patients- the OSAS group – 27 patients with OSAS (apnea/hypopnea index, AHI>5) including the subgroup of 11 patients with severe OSAS (AHI ≥ 30), from patients without OSAS (AHI<5) who served as a control group (n = 11). The third group (CPAP-OSAS group) involved OSAS patients (n = 22), successful at the first attempt of CPAP treatment (Table 1, 2, and 3).

**Table 1:** Demographics of investigated groups

	Subjects that underwent polysomnography			
	
Parameter	OSAS group, n = 27	Severe OSAS subgroup, n = 11	CPAP-OSAS group, n= 22	Controls, n = 11
Male/Female	25/2	10/1	17/5	9/3

Smokers	15	0	15	6

Age [yrs]	53 ± 13	55 ± 15	58 ± 8	50 ± 10

BMI [kg/m^2^]	31.1 ± 5.1	32.1 ± 6.5	34.3 ± 7.3	28.8 ± 5.5

NC [cm]	42.6 ± 2.6	43.7 ± 2.9	43.7 ± 4.1	41.5 ± 2.0

ESS	11 ± 1	11 ± 5	15 ± 1*	9 ± 1

TST [h]	4.8 ± 1.2	4.7 ± 1.3	5.2 ± 1.6	5.3 ± 1.0

AHI [n/h]	31 ± 5	58 ± 18 †	9 ± 2*	2 ± 1*

ODI [n/h]	31 ± 5	55 ± 19 †	9 ± 3*	2 ± 1*

Mean SaO_2 _[%]	87 ± 1	84 ± 5 †	89 ± 1	91 ± 1*

Min SaO_2 _[%]	74 ± 3	68 ± 15 †	81 ± 2	88 ± 1*

T_SaO2_<88% [min]	55 ± 12	99 ± 74 †	12 ± 4*	2 ± 1*

Snoring [%TST]	29 ± 24	28 ± 24	10 ± 14*	20 ± 25

OSAS – patients with untreated OSAS; Severe OSAS – patients with AHI ≥ 30; CPAP-OSAS – patients with OSAS treated successfully at a first attempt with CPAP, controls – subjects without OSAS; BMI – body mass index; NC – neck circumference; ESS – Epworth sleepiness score; TST – total sleep time; AHI (apnea/hypopnea index) – the number of apneas and hypopneas per hour of sleep; ODI (oxygen desaturation index) – the number of desaturations ≥ 4% per hour of sleep; SaO_2_-nocturnal oxygen saturation; T_SaO2 _– duration of nocturnal oxygen saturation < 88%. Polysomnography data expressed as a mean ± SD. The daily cigarette consumption was 19 ± 12, 21 ± 13, and 17 ± 6 for current cigarette smokers in OSAS, CPAP-OSAS and control groups respectively. Blood for chemiluminescence measurement and other determinations was taken before and after polysomnographic controlled sleep.

* – p < 0.05 vs. OSAS group, † – p < 0.05 vs. CPAP-OSAS and controls groups.

Selected baseline characteristics of CPAP-OSAS patients before treatment initiation were: AHI 58 ± 29; ODI 56 ± 26; mean SaO_2 _82 ± 7 %, min SaO_2 _75 ± 9 %, and T_SaO2 _< 88% 105 ± 90 min. of 5.2 ± 1.7 h TST.

**Table 2:** Comorbidity in the investigated patient groups

Disease	Number of patients with a given disease		
	
	OSAS n = 27	CPAP-OSAS n = 22	Controls n = 11
Arterial hypertension	16	16	4

Ischemic heart disease	7	7	2

Diabetes	2	4	0

Gout	0	4	0

OSAS – patients with untreated OSAS; CPAP-OSAS – patients with OSAS treated successfully after a first attempt with CPAP, controls – subjects without OSAS.

**Table 3:** Ongoing pharmacological treatment in studied groups

Pharmacological treatment	Number of patients receiving treatment		
	
	OSAS, n = 27	CPAP-OSAS, n = 22	Controls, n = 11
ACEI	12	10	2

Diuretics	11	6	1

Ca^2+ ^channel blocker	4	5	1

Beta-blockers	4	1	1

Nitrates	4	4	1

Digitalis	3	1	0

Statins	8	6	0

Allopurinol	0	4	0

Ticlopidine	2	0	1

Gliclazide	1	5	0

ACEI – angiotensin converting enzyme inhibitors; OSAS – patients with untreated OSAS; CPAP-OSAS – patients with OSAS treated successfully after a first attempt with CPAP, controls – subjects without OSAS. Four, 2, and 6 subjects were free of any medication in OSAS, CPAP-OSAS and controls group, respectively.

### Study Design

All participants were admitted to the Sleep Laboratory at approximately 8:30 p.m., previously instructed to consume a light final meal before 7:00 p.m. Venous blood was collected twice for blood cell count and measurement of oxidative stress markers – before and after polysomnography-controlled sleep, at about 9:30 p.m. and 6:00 a.m. (just after wakening up), respectively. Nine ml of venous blood were drawn into sodium heparin vacuette tubes (placed in ice-cold water) and EDTA-K_3 _vacuette-tubes (Greiner bio-one GmbH, Kremsmunster, Austria). The heparinized blood was used for measuring whole blood H_2_O_2 _activity, followed by its addition to other reagents within 10 seconds from collection. Luminol enhanced whole blood chemiluminescence (LBCL) measurement procedure (EDTA-K_3 _blood samples with determined blood cell count) began no later than 30 min. following blood collection. Plasma samples obtained from EDTA-K_3 _blood (30 min incubation at 37°C, subsequent centrifugation for 10 min at 1500·g and 4°C) were stored at -70°C for no longer than 3 weeks until FRAP determination. Blood cell count was measured with Micros Analyzer OT 45 (ABX, Montpellier, France). In the event of an at night awakenings, only plain mineral water was allowed to be drunk *ad libitum*. The Medical University of Lodz Ethics Committee approved the study protocol and all participants provided a written, informed consent.

### Luminol enhanced whole blood chemiluminescence assay

A luminol enhanced whole blood chemiluminescence (LBCL) technique was employed as a measure of resting and agonist induced ROS production by circulating phagocytes [17,18] in order to avoid any priming and/or activation of oxidative cell response due to isolation procedures [18]. Moreover, to avoid any possible bias related to patients' interindividual variability and differences in comorbidity and pharmacological treatment, LBCL was measured before (evening) and just after polysomnography-controlled sleep (morning) in a matched manner. Therefore, evening results served as reference values designed for morning data, presumably affected by apnea/hypopnea episodes in addition to subsequent IH during sleep.

LBCL as a measure of resting and fMLP-stimulated circulating phagocytes (PMNs and monocytes) ability to produce ROS was determined according to Kukovetz et al. [17] with some modifications [22]. Briefly, 3 μl of blood sample was added to 947 μl of mixture solution (composed of 1 ml sterile Ringer solution, 200 μl 5% D-glucose solution, 3.6 ml deionized water and 5 ml 1.4 M luminol solution) which was pre-warmed in darkness up to 37°C for 60 min. The samples were placed in the 1251 luminometer (Bio-Orbit, Turku, Finland) and incubated for 30 min. at 37°C. Afterwards the resting chemiluminescence was recorded continuously for 1 min. and then 50 μl fMLP solution was added automatically to a final concentration of 0.02 mM, with continuation of the light emission measurement for an additional 7 min. All individual results were obtained as a mean of four measurements with LBCL parameters assessing resting chemiluminescence (rCl) – the average resting chemiluminescence prior to the addition of fMLP; peak chemiluminescence (pCL) – maximal chemiluminescence signal after the addition of fMLP. Total light emission (tCL) – the area under the chemiluminescence intensity curve after the addition of fMLP until its return to baseline and peak time – the time (seconds) from the addition of fMLP to the appearance of pCL were also assessed. rCL and pCL were expressed in arbitrary units (aU) per 10^4 ^phagocytes (PMNs and monocytes) present in the assayed sample, while tCL in aU·s/10^4 ^phagocytes. Preliminary experiments with platelet rich plasma excluded the contribution of platelets to fMLP-evoked LBCL (data not shown).

### The H_2_O_2 _activity in the whole blood

H_2_O_2 _activity was measured in whole blood using the phenol red oxidation method [19,23] with some modifications. Briefly, 600 μl of blood was either added to 60 μl 0.2 M NaN_3 _solution or 60 μl of bovine catalase solution (3000 I.U. per sample) in 0.9% NaCl. Both tubes were subsequently incubated for 5 min at 37°C and then a 30 μl 0.028 M solution of phenol red in deionized water with a 30 μl (27.75 I.U. per sample) of HRP solution in 50 mM phosphate buffer (pH = 7.0) were then added to each test-tube. Afterwards, the samples were incubated for 10 min at 37°C and then centrifuged (10 min., 1500·g). One hundred μl of supernatant was transferred into a cuvette containing 900 μl PBS and 10 μl 1 M NaOH reading (spectrophotometer Ultrospec III, LKB Biochrom England) its absorbance at 610 nm (A_610_). The calibration curve was made with 600 μl samples of standard H_2_O_2 _solutions (increasing concentrations from 0.1 to 12 μM, 13 concentration points) in PBS added to 60 μl 0.2 M NaN_3 _solution and subsequently processed in the same way as blood specimens. The concentration of H_2_O_2 _(μM) in the blood specimens was calculated according to the regression equation: y = 33.12(x_1_-x_2_) – 0.23 (r = 0.97, p < 0.001) where y was the H_2_O_2 _concentration and x_1_-x_2 _was the difference between the A_610 _of a sample with NaN_3 _(x_1_) and A_610 _of a sample with catalase (x_2_) being the blank (all H_2_O_2 _was decomposed by the enzyme). The method sensitivity was 0.25 μM of H_2_O_2_. Individual results were obtained as the mean of two measurements.

### Ferric reducing ability of plasma

FRAP ascertainment was measured following the procedure originally described by Benzie and Strain [20] with some modifications [24], in which Fe^3+ ^to Fe^2+ ^ion reduction at low pH caused the formation of a coloured ferrous-TPTZ complex, resulting in an increase in absorbance at 593 nm (A_593_). Briefly, 30 μl of plasma was mixed with 90 μl of deionized water and then added to 900 μl of a FRAP reagent (pre-warmed to 37°C) while sample absorbance at 593 nm was continuously measured over 8 min. at 37°C (Ultrospec III, with a Spectro-Kinetics software). Control samples (blank) received 120 μl of water. FRAP reagent was prepared just before the assay by adding in the following order: 10 ml 300 mM acetate buffer (pH 3.6), 1 ml 10 mM TPTZ in 40 mM HCl, and 1 ml 20 mM aqueous FeCl_3 _solution. Calibration was performed with a FRAP reagent containing the addition of FeCl_2 _(total sample volume 1.02 ml, final concentrations from 20 to 2000 μM, 9 concentration points). Absorbance was linear (r = 0.98, p < 0.001). Intra- and inter- assay coefficients of variations tested on 10 aliquots of pooled plasma from 10 healthy donors were less than 8%. Individual results were obtained from duplicate experiments and were expressed as a concentration of Fe^3+ ^ions reduced into Fe^2+ ^after 0, 1, 2, 3, 4, 5, 6, 7 and 8 min. incubation of plasma sample with FRAP reagent. Calculations were done according to the formula: Y [μM] = 1687.9 X – 3.3, where Y – concentration of Fe^3+ ^reduced ions, X – difference between A_593 _(assayed sample) and A_593 _(blank).

### In vitro effect of H_2_O_2 _on FRAP

Nine hundred and fifty μl of pooled plasma samples (obtained from 10 healthy donors) were mixed with 50 μl of deionized water or 50 μl of various H_2_O_2 _solutions and were incubated for 5, 30 and 60 min at 37°C. The final concentration of H_2_O_2 _in plasma samples were 0 μM, 47 μM, 16.5 mM, and 82.5 mM, respectively. Then the samples were centrifuged (5 min., 1500·g, 4°C) and 30 μl of plasma after mixing with 90 μl of water was added to 0.9 ml of FRAP reagent. A_593 _was recorded over a 10 min. incubation period at 37°C while the concentration of reduced Fe^3+ ^ions was calculated as above.

### Statistical analysis

All data were expressed, dependent on the distribution, as the mean and standard deviation (SD) and/or median and quartile range. Shapiro-Wilk W test was used for normality testing. The differences between groups (normal data) were assessed using an analysis of variance for independent variables. Repeated measures ANOVA was applied for dependent variables (evening vs. morning). For data not normally distributed, the Kruskal-Wallis test and Friedman test were used. In the case of significance, appropriate post-hoc tests were implicated. An additional comparison between two selected parameters with an abnormal distribution was conducted using the Wilcoxon test (for dependent variables) and the U Mann-Whitney test (for independent variables). The t-student test for dependent variables was used in comparison of two normally distributed parameters. A p value of < 0.05 was considered significant. For differences between means, the 95% confidence intervals were calculated.

## Results

### White blood cells, phagocytes and hematocrit

In all three investigated groups, morning white blood cell counts were lower as compared to the evening counts (p < 0.05). Evening and morning quantities of PMNs collectively with monocytes differed significantly (p < 0.05). Indistinguishable manifestations arose in the subgroup of 11 patients with severe OSAS (data not shown). This explains why the LBCL was expressed per 10^4 ^phagocytes despite the relatively diminutive time-interval between evening and morning blood collections, in addition to the data analysis of LBCL as a dependent variable. Conversely, hematocrit was significantly unchanged (p > 0.05) (Table 4). Therefore, correction of plasma volume changes in FRAP and H_2_O_2 _activities in the morning data was unnecessary.

**Table 4:** White blood cells, phagocytes and hematocrit

		OSAS	CPAP-OSAS	CONTROLS
Hematocrit [%]	Evening	39.2 ± 3.8	40.8 ± 4.8	37.4 ± 5.5
	
	Morning	38.6 ± 4.9	40.5 ± 6.1	38.6 ± 5.7
	
	Diff & 95% CI	0.6 (-0.78–1.95)	0.3 (-0.74–1.38)	1.2 (0.42–1.99)

WBC [10^3^/μl]	Evening	8.06 ± 2.00	8.01 ± 2.30	8.61 ± 2,00
	
	Morning	7.20 ± 1.91	7.26 ± 2.23	7.67 ± 2.92
	
	Diff & 95% CI	0.86 (0.86–1.18)	0.75 (0.75–1.29)	0.94 (0.46–1.65)

PMNs+monocytes [10^3^/μl]	Evening	5.68 ± 1.67	5.75 ± 2.00	5.50 ± 2.26
	
	Morning	5.13 ± 1.63	5.19 ± 1.82	4.67 ± 1.45
	
	Diff & 95% CI	0.55 (0.32–0.80)	0.56 (0.15–0.98)	0.83 (0.31–1.36)

Data expressed as a mean ± SD (standard deviation)

WBC – white blood cells, PMNs – polymorphonuclear leukocytes, 95% CI – 95% confidence interval, Diff – difference of means with 95% confidence interval

### Luminol enhanced whole blood chemiluminescence

No analyzed parameters of LBCL (rCL, pCL, tCL and peak-time) altered significantly after sleep in patients with OSAS and controls (Table 5). There were also no significant differences between all three of the investigated groups in respect to these parameters. Unpredictably, in all groups, the tendency (although, insignificant) to present a decreased LBCL (rCL, pCL and tCL) subsequent to sleep was prominent.

**Table 5:** Luminol enhanced whole blood chemiluminescence (LBCL) measured before and after polysomnography controlled sleep

		Patients undergoing polysomnography			
		
Chemiluminescence parameter	Time of the day	OSAS	Severe OSAS	CPAP-OSAS	Controls
rCL [aU/10^4^p]	Evening	2.4 ± 2.2	1.9 ± 1.8	2.2 ± 1.9	1.9 ± 1.8
	
	Morning	2.3 ± 2.2	2.5 ± 2.8	1.5 ± 0.9	1.6 ± 1.5
	
	Diff and 95% CI	0.1 (-0.7 – 0.8)	0.6 (0.15 – 1.02)	0.7 (0.1 – 1.2)	0.2 (-0.1 – 0.5)

pCL [aU/10^4^p]	Evening	6.4 ± 5.1	4.9 ± 4.7	6.0 ± 4.4	5.5 ± 4.3
	
	Morning	5.9 ± 4.6	5.9 ± 4.8	4.3 ± 2.9	4.7 ± 2.5
	
	Diff and 95% CI	0.5 (-0.9 – 1.8)	0,9 (-0.4 – 2.2)	1.6 (0.4 – 2.9)	5.2 (-2,0 – 12.4)

tCL [aU·s/10^4^p]	Evening	1939 ± 1532	1503 ± 1391	1805 ± 1278	1642 ± 1316
	
	Morning	1790 ± 1371	1778 ± 1442	1313 ± 820	1416 ± 961
	
	Diff and 95% CI	149 (-275 – 573)	275 (-117 – 668)	492 (132 – 852)	225 (22 – 430)

Peak time [s]	Evening	281 ± 31	276 ± 37	293 ± 30	270 ± 24
	
	Morning	275 ± 30	277 ± 37	284 ± 22	339 ± 23
	
	Diff and 95% CI	6 (0.3 – 11.5)	1 (-5.3 – 6.5)	9 (0.1 – 16.3)	69 (-22 – 160)

rCl – the average resting chemiluminescence prior to addition of fMLP; pCL – maximal chemiluminescence signal after addition of fMLP; tCl – total light emission after cell stimulation with fMLP; peak time – the time from fMLP addition to the appearance of pCL; OSAS – patients with the untreated OSAS; Severe OSAS – patients with AHI ≥ 30; CPAP-OSAS – patients with OSAS treated successfully after a first attempt with CPAP, controls – subjects without OSAS Results expressed as mean ± SD, difference of means with 95% confidence interval (Diff and 95% CI) No significant differences (p > 0.05) were found for evening and morning LBCL parameters within and between the studied groups.

Furthermore, patients with severe untreated OSAS (n = 11) did not reveal any differences between evening and morning LBCL (p > 0.05). However, in the subgroup, the observance of the reverse tendency to increase morning LBCL parameters is notable (Table 5).

### Ferric reducing ability of plasma

**Figure 1:**
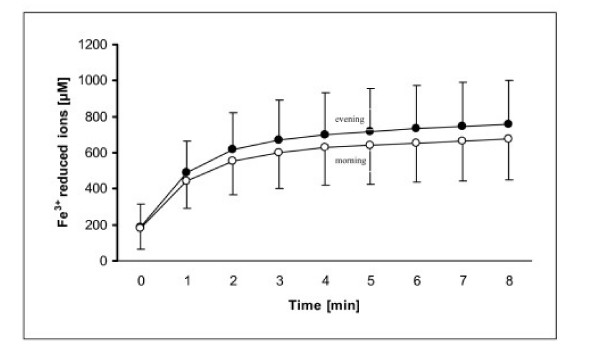
**Ferric reducing ability of plasma (FRAP) before and after polisomnography controlled sleep**. Closed circles – evening (before polisomnography controlled sleep). Open circles – morning (after polisomnography controlled sleep). Results of FRAP in 27 subjects with OSAS expressed as mean and standard deviations represent the concentration of Fe^3+ ^reduced ions after 0, 1, 2, 3, 4, 5, 6, 7 and 8 min incubation of patient plasma sample with the FRAP reagent. No significant differences (p > 0.05) were found for evening and morning FRAP at all incubation time-points.

The 3 min. incubation of a plasma sample with a FRAP reagent (containing Fe^3+ ^and TPTZ) is recommended in receiving the most reliable results in plasma antioxidant activity [20]. Table 6 shows this data after 3 min. incubation with no significant differences noted between evening and morning plasma FRAP within all of the analyzed groups. There were also no significant differences between the corresponding values found in OSAS, CPAP- OSAS, as well as the control groups. FRAP reflects the sum of antioxidant activities in various compounds [20]. They may encompass dissimilar potentials in reducing Fe^3+ ^ions, thereby varying along with the time of sample incubation in their contribution to FRAP. Therefore, we additionally analyzed the effect of apneas-induced IH events on FRAP obtained after various times of incubation. Figure 1 shows the mean evening and morning FRAP of OSAS patients obtained after plasma sample incubation from 0 till 8 min. No significant differences between the evening and morning FRAP values were evident in each of the times during incubation. Similar results were obtained in CPAP-OSAS subjects, control group and the subgroup of severe OSAS patients (data not shown). In vitro experiments revealed that the incubation of plasma with H_2_O_2 _at concentrations: 47 μM and 16.5 mM for 1 hour did not suppress FRAP (data not shown). Even H_2_O_2 _at a concentration of 82.5 mM did not significantly decrease FRAP (587 ± 12 μM vs. 603 ± 16 μM, n = 5, p > 0.05).

**Table 6:** Ferric reducing ability of plasma (FRAP) before (evening) and after (morning) polysomnography controlled sleep

Patients group	FRAP [μM] measured at		Diff, 95% CI
		
	Evening	Morning	
OSAS, n = 27	671 ± 221	602 ± 202	70 (7–133)

Severe OSAS, n = 11	729 ± 156	650 ± 170	79 (37–122)

CPAP-OSAS, n = 22	597 ± 199	591 ± 242	7 (-44–57)

Controls, n = 11	634 ± 229	597 ± 201	37 (-3–77)

OSAS – patients with the untreated OSAS; CPAP-OSAS – patients with OSAS treated successfully after a first attempt with CPAP, controls – subjects without OSAS. Results expressed as a mean ± SD represent concentration of Fe^3+ ^reduced ions after 3 min. incubation of plasma sample with the FRAP reagent. Diff, 95% CI – difference of means with 95% confidence interval

No significant differences (p > 0.05) were found for evening and morning FRAP within and between the studied groups.

### H_2_O_2 _activity in the whole blood

Only eight OSAS patients had detectable H_2_O_2 _levels in the whole blood in the evening, while seven OSAS patients observed detectable H_2_O_2 _post awakening. In the whole OSAS group, evening H_2_O_2 _– 0.23 ± 0.44 μM (0; 0.30) did not differ (p > 0.05, n = 27) from the morning – 0.22 ± 0.61 μM (0; 0.40) (95% CI -0.12 – 0.13). In the subgroup of patients with severe OSAS, the ratio of positive H_2_O_2 _readings were 4/11 and 2/11 for the evening and morning measurements. For CPAP-OSAS and the control group, the ratio of H_2_O_2 _positive results were 4/22 and 3/11 for both evening and morning blood samples, respectively. Similar to the whole group of OSAS patients, no differences were found between the morning and evening H_2_O_2 _activities in the remaining two groups as well as in the subgroup of patients with severe OSAS (data not shown). Furthermore, no differences manifested among all three groups (data not shown).

## Discussion

Parameters of resting and fMLP-induced LBCL were the co-primary variables in our study. The morning and evening chemiluminescence parameters like rCL, pCL and tCL as well as FRAP were higher in OSAS patients in comparison with controls. Nevertheless, we did not find any differences between morning and evening LBCL parameters between and within the groups of OSAS, CPAP-OSAS and the matched controls. Moreover, LBCL did not rise significantly after polysomnography controlled sleep in the subgroup of patients with severe untreated OSAS. Therefore, our results suggest that OSAS related IH did not enhance ROS production by blood PMNs and monocytes. This finding is in contrast with the results of two previous studies showing increased ROS production from isolated PMNs (following stimulation with fMLP) and some subpopulations of whole blood monocytes (resting and phorbol myristate acetate – activated) of OSAS patients [14,15].

Apart from the absence of cell isolation procedures and the usage of monoclonal antibodies to surface cell markers possibly altering PMNs and monocytic responsiveness to agonist stimulation, there were other significant differences between the protocols of our study and the aforementioned. In previous studies, the control subjects were not matched to OSAS patients in respect to age, BMI, cigarette smoking habits in addition to comorbidity [14,15]. Moreover, both investigations did not provide information concerning concomitant pharmacologic treatment [14,15]; furthermore, experiments on the effect of OSAS related IH on monocytic activity were based on only 8 to 10 subjects out of the 18 enrolled [15] devoid of an unambiguous selective criterion.

In view of the fact that the majority of OSAS patients are obese often developing a variety of cardiovascular and metabolic diseases [1,2], there exists a challenge in finding appropriate control subjects, chiefly in respects to comorbidity and concomitant pharmacological treatment. In our study, we overcome this impediment by measuring LBCL prior to and following polysomnographic controlled sleep, formulating comparisons within and between study groups. These approaches act to possibly eliminate biases related to patient medication, intensifying the quality of our results in showing no priming or activation of blood phagocytes in untreated OSAS patients.

The sympathetic over activity in OSAS patients resulting in the rise of plasma norepinephrine and epinephrine levels [25-27] may perhaps be responsible in the insignificant changes between evening and morning blood LBCL in OSAS patients. Reports have demonstrated a higher morning (following awakening) plasma norepinephrine concentration than those before sleep in OSAS patients by 24% [25]. Nonetheless, a night of successful CPAP therapy resulted in a down-regulation of sympathetic activity as well as a decrease in circulating catecholamines by 20% [26]. Catecholamines in vitro, especially epinephrine at physiologic levels revealed concentration dependent inhibition of fMLP-induced degranulation and superoxide radical production by human PMNs [28-30]; with a capability of suppressive monocytic activity as well [31,32]. Therefore, exposition of circulating phagocytes to increased concentrations of catecholamines may result in their insusceptibility to the priming effect of IH in respiratory burst, assuming responsibility for the negative results in untreated OSAS patients. On the other hand, the observation of rapidly reversible sympathetic over-activation due to successful treatment [26] elucidates the indifferences among morning and evening LBCL in CPAP-OSAS group. This may result from the simultaneous reduction of IH episodes along with plasma catecholamine suppression, together with a rapid turnover of circulating PMNs; strongly supported by a recent study demonstrating the inhibitory effect of exercise on hypobaric hypoxia-induced enhancement of ROS production by PMNs in healthy volunteers [33]. It cannot be excluded that our OSAS patients encountered an inadequate amount of apneas/hypopneas, consequently observing insufficient blood desaturations to prime PMNs to fMLP stimulation. In the abovementioned study, overnight hypobaric hypoxia decreased the average SaO_2 _from a baseline of 98% to 93% concluding a high altitude stay [33].

A decreased average SaO_2 _(84%) along with minimal SaO_2 _(68%) was evident in our investigated patients, particularly those with severe OSAS, due to numerous transient desaturations. The activity of two secondary variables (FRAP and H_2_O_2 _activity in the whole blood) in OSAS patients after sleep was compatible to the results of LBCL. We did not observe sleep-induced ROS overproduction in the blood (LBCL, H_2_O_2 _activity) of untreated OSAS patients, therefore no suppression of morning FRAP was noted. On the other hand, resistance of FRAP to 1 h in vitro incubation with high concentrations of H_2_O_2 _suggests strong antioxidant plasma capacity. Therefore, FRAP suppression related to significant expenditure of circulating antioxidants with high Fe^3+ ^reducing activity will occur in vivo in the case of large and long (probably longer than one night) systemic ROS overproduction.

The results of our study suggest that circulating phagocytes (PMNs and monocytes) are not the main culprit of OSAS consequences in the human body. It does not exclude augmented ROS production and activation of the systemic ROS signaling [7,14,34]. Circulating phagocytes probably do not take part in oxidative stress, which does not synonymously reject the oxidative stress presence in OSAS patients. It can take place near or exactly in blood vessel endothelium, which can significantly accelerate atherosclerosis.

## Conclusion

In conclusion, untreated OSAS patients did not present with elevated resting and fMLP induced LBCL when compared with age-, BMI-, and smoking habits- matched controls. Moreover, no significant alterations of evening vs. morning LBCL, blood H_2_O_2 _activities and FRAP were noted in OSAS patients. These indicate that circulating PMNs and monocytes did not produce increased amounts of ROS and did not contribute to oxidative stress in OSAS patients.

## Competing interests

The authors declare that they have no competing interests.

## Authors' contributions

IGK organized the whole study, prepared the solutions for laboratorial experiments, carried out morphology, FRAP, LBCL and H_2_O_2 _activity in the whole blood measurement, analyzed all patients histories and all experiments results, prepared the manuscript. AK presented the idea and the aim of the study to our patients, carried out all polysomnographies, collected blood samples and participated in LBCL and H_2_O_2 _activity in the whole blood measurement. PB took patients histories and described about 50 % polysomnographies. Based on them he diagnosed patients and, in case of need, chose CPAP with adequate pressure. MKr took patients histories, described about 50 % remaining polysomnographies, based on them diagnosed patients and also, in case, chose CPAP with adequate pressure. GE carried out the in vitro experiment verifying the effect of H_2_O_2 _on FRAP. MKa performed the statistical analysis. DN conceived of the study, and participated in its design and coordination. He also drafted the manuscript and had decisive influence on the discussion course. All authors read and approved the final manuscript.
